# Changing ethnic inequalities in mortality in New Zealand over 30 years: linked cohort studies with 68.9 million person-years of follow-up

**DOI:** 10.1186/s12963-017-0132-6

**Published:** 2017-04-26

**Authors:** George Disney, Andrea Teng, June Atkinson, Nick Wilson, Tony Blakely

**Affiliations:** University of Otago, 23a Mein Street, Wellington, New Zealand

**Keywords:** Ethnicity, Mortality, Inequality, Indigenous, Typology, Cardiovascular disease, Cancer, Māori, Pacific peoples, New Zealand

## Abstract

**Background:**

Internationally, ethnic inequalities in mortality within countries are increasingly recognized as a public health concern. But few countries have data to monitor such inequalities. We aimed to provide a detailed description of ethnic inequalities (Māori [indigenous], Pacific, and European/Other) in mortality for a country with high quality ethnicity data, using both standard and novel visualization methods.

**Methods:**

Cohort studies of the entire New Zealand population were conducted, using probabilistically-linked Census and mortality data from 1981 to 2011 (68.9 million person years). Absolute (standardized rate difference) and relative (standardized rate ratio) inequalities were calculated, in 1–74-year-olds, for Māori and Pacific peoples in comparison to European/Other.

**Results:**

All-cause mortality rates were highest for Māori, followed by Pacific peoples then European/Other, and declined in all three ethnic groups over time. Pacific peoples experienced the slowest annual percentage fall in mortality rates, then Māori, with European/Other having the highest percentage falls – resulting in widening relative inequalities.

Absolute inequalities, however, for both Māori and Pacific males compared to European/Other have been falling since 1996. But for females, only Māori absolute inequalities (compared with European/Other) have been falling.

Regarding cause of death, cancer is becoming a more important contributor than cardiovascular disease (CVD) to absolute inequalities, especially for Māori females.

**Conclusions:**

We found declines in all-cause mortality rates, over time, for each ethnic group of interest. Ethnic mortality inequalities are generally stable or even falling in absolute terms, but have increased on a relative scale. The drivers of these inequalities in mortality are transitioning over time, away from CVD to cancer and diabetes; such transitions are likely in other countries, and warrant further research. To address these inequalities, policymakers need to enhance prevention activities and health care delivery, but also support wider improvements in educational achievement and socioeconomic position for highest need populations.

## Background

Internationally, ethnic and indigenous inequalities in mortality within countries are increasingly recognized as a public health concern [1–4]. But few countries have data to monitor such inequalities accurately [5]. We aimed to provide a detailed description of ethnic inequalities (Māori [indigenous] and Pacific compared to the benchmark, European/Other) in mortality for a country with high-quality ethnicity data, using both standard methods and novel visualization methods.

Ethnic inequalities in mortality in New Zealand are well-documented [6]. Measurement of these inequalities matter, as they are markers of social (in)justice and (in)equity [3]. Furthermore, understanding what causes these inequalities in the past, and how they are changing over time, with qualitative (at least) projections into the future, highlight where future mortality reduction gains could be made by ethnic group and inequalities reduced [3].

There is some debate as to whether health inequality should be measured on relative or absolute scales [7]. We demonstrate, however that, alongside the trend in the mortality rate, it may be optimal to consider both scales of inequality simultaneously, to provide a more holistic analysis of population mortality trends.

The indigenous New Zealand Māori population have had worse health than the European population since colonization during the 1800s. Whilst, life expectancy and mortality gaps have closed since c. 1900 [8], with a notable period of widening again in the 1980s and 1990s [6], in part driven by structural changes to liberalize the economy that impacted harder on Māori and probably also in part due to delayed phasing of the cardiovascular disease (CVD) epidemic for Māori compared to European/Other [9], there was still a seven-year gap in life expectancy in 2011 [10]. Reasons are numerous and intertwined: for example, the ongoing structure of domination and inequities associated with colonization [11], which also plays a key structuring role in the ethnic patterning of risk factors (higher tobacco smoking rates for Māori, for example), and worse access to health services [8], for example.

Pacific peoples began migrating to New Zealand in substantial numbers after World War II. Since reliable mortality data existed (from c. 1980 onwards), Pacific mortality has been intermediary between European/Other and Māori – despite, on average, lower socioeconomic position of Pacific than Māori, and often healthier risk factor profiles (e.g., Pacific peoples (depending on country of origin) generally have lower smoking rates than Māori and often also European/Other [12]).

Monitoring social group (whether this is ethnic group, gender group, or socioeconomic group) differences in health are important. For example, they inform us about the impacts of structural inequalities and drivers of health in society [13]. The assembly and analysis of the best data available on mortality and ethnicity is one important way that we can deepen understanding of how health differences between social groups change over time and may be preventable.

Internationally, a major limitation of investigations into ethnic inequalities in health has been the quality of data on ethnicity [14, 15]. In particular, the use of surrogate indicators of ethnicity, such as country of birth or observer-assigned ethnicity, are often used instead of self-reported ethnicity on mortality data, which can lead to an mis-estimation (often underestimation) of mortality rates for indigenous groups [5], when using census (or other population-wide count data) as a denominator.

Extensive efforts have been made in New Zealand to improve information on ethnicity in health datasets. However, issues remain with consistency and recording [16]. These limitations are circumvented in this paper, through the use of the New Zealand Census Mortality Study (NZCMS). Information on people’s self-identified ethnicity is collected through the Census. Census data is then linked to mortality records, at the individual level, creating repeated cohort studies of the entire population. Ethnicity from the Census file only is used for the calculation of mortality rates, thus avoiding numerator-denominator bias [17].

In this study we build on our previous work by presenting a detailed analysis of both changing relative and absolute inequalities in mortality rates for both Māori and Pacific peoples (compared to the European/Other ethnic group), over the last 30 years. Specific objectives of the study were:

- To present trends in all-cause and cause-specific (CVD, lung cancer, non-lung cancer, respiratory disease, diabetes, unintentional injury and suicide) mortality by ethnic group.
- To present trends in mortality inequalities for Māori and Pacific each compared to European/Other, including using a new holistic graphical visualization tool.
- To comment on possible reasons for these inequalities and trends, and project what the future might hold.

## Methods

### Assembly of data

We individually and probabilistically linked mortality and census records [18]. Mortality records from 1981–1999 (the first four cohorts) for people who died within three years of the previous census night (1981, 1986, 1991, and 1996 respectively) were assembled and linked to the census. We limited our follow-up period to three years, in the first four cohorts due to concerns about diminishing record linkage. For the period 2001–2011 all mortality records were assembled, with little diminishing linkage rates as the follow-up period progressed. Direct age standardization, using the World Health Organization standard, was used to calculate rates, standardized rate differences (SRDs), and rate ratios (SRRs). All the data used in this report (and further disaggregations by mortality, age, and socioeconomic status are publicly available at the NZCMS Data Explorer [19]).

### Ethnicity, mortality.,and age variables

The ethnic groups of interest were selected based on a consideration of statistical power and relevance to the main aim of monitoring ethnic inequalities in mortality. Information on ethnicity was taken from the Census file only. A total response ethnicity approach was used for Māori and Pacific peoples; the Total Asian group was omitted due to lack of statistical power when disaggregated by the mortalities of interest. Using “total response” means that there can be a slight overlap, where people self-identify as both Māori and Pacific [20].

The remaining people (who did not identify as Māori, Pacific, or Asian) were assigned as European/Other; which, was set as the reference category. As mentioned previously, people of European background, on average, have had lower mortality rates since colonization, so setting this group as the benchmark population, makes conceptual sense.

The eight mortalities (including all-cause) with the highest standardized rates for the whole population, in the most recent (2006) cohort, was selected for analysis. Mortalities were coded using the International Classification of Disease (ICD-9 and ICD-10 in square brackets): all-cause, cardiovascular disease (393-438, 440-459 *[I05-I15, I20-I99]*), lung cancer (162 *[C34]*), non-lung cancer (140-161, 163-209 *[C00-C32, C35-C97]*), respiratory disease (470-478, 490-519 *[J30-J39, J40-J47, J60-J70, J95-J99]*), diabetes (250 *[E10-E14]*), unintentional injury (800-949 *[V01-X59]*), and suicide (950-959, 980-989 *[X60-X84]*).

We restricted our analysis to 1-74-year-olds. To be linked to a census record, individuals in the mortality data needed to be born prior to census night, i.e., it is a closed cohort. Accordingly, these cohorts are not well-positioned to examine infant mortality.

### Annual percentage change, tests for linear trend, and contribution to absolute inequality

Poisson regression models were fitted on age-standardized mortality rates, for 1-74-year-olds across all cohorts, with the mid-date of each follow-up period specified as the independent variable. The estimated coefficient from the model fit was then transformed onto the linear scale, and converted into an annual percentage change in mortality. These values are displayed in Tables 1 and 2. We have also calculated p-values testing whether there are linear trends in rates differences, and the log of standardized rates and rate ratios. However, not all of the trends we present exhibit a linear pattern across all six cohorts, but still do change significantly. We therefore also refer to significant non-linear changes between cohorts for given causes of death, if the 95% confidence intervals between the two cohorts of interest do not overlap.

**Table 1:** 1–74-year-old male all-cause and cause-specific standardized mortality rates (per 100,000), standardized rate differences (per 100,000), standardized rate ratios, annual percentage change, and p for linear trend statistics

Males		Total Māori			Total Pacific			European/Other
Follow-up period	Mortality	Standardized rate (per 100,000)	Standardized rate difference (per 100,000)	Standardized rate ratio	Pacific	Standardized rate difference	Standardized rate ratio	Standardized rate (per 100,000)
1981–1984	All-cause	1005 (944 - 1066)	443.1 (381.1 - 505.1)	1.79 (1.68 - 1.91)	671 (561 - 780)	108.7 (-1.1 - 218.6)	1.19 (1.01 - 1.41)	562 (553 - 571)
1986–1989		922 (869 - 975)	405.8 (352.1 - 459.6)	1.79 (1.68 - 1.90)	784 (690 - 878)	267.3 (172.9 - 361.6)	1.52 (1.34 - 1.71)	516 (508 - 525)
1991–1994		960 (908 - 1011)	513.8 (461.7 - 565.8)	2.15 (2.03 - 2.28)	630 (556 - 703)	183.9 (110.3 - 257.5)	1.41 (1.26 - 1.59)	446 (438 - 454)
1996–1999		898 (858 - 937)	521.3 (481.3 - 561.2)	2.39 (2.27 - 2.50)	679 (620 - 738)	302.4 (243.3 - 361.5)	1.80 (1.65 - 1.97)	376 (369 - 383)
2001–2006		806 (778 - 835)	467.1 (438.0 - 496.3)	2.38 (2.29 - 2.47)	598 (559 - 638)	259.3 (219.4 - 299.2)	1.76 (1.65 - 1.89)	339 (334 - 344)
2006–2011		701 (676 - 725)	424.3 (399.4 - 449.2)	2.53 (2.44 - 2.63)	495 (461 - 528)	218.1 (184.6 - 251.5)	1.79 (1.67 - 1.92)	276 (272 - 281)
Change p.a. (%)		-1.21			-1.19			-2.65
P Trend		<0.01	0.6	<0.01	0.02	0.83	0.04	<0.01
1981–1984	CVD	461 (419 - 503)	197.2 (154.5 - 239.8)	1.75 (1.59 - 1.92)	265 (198 - 333)	1.7 (-65.7 - 69.1)	1.01 (0.78 - 1.30)	264 (258 - 270)
1986–1989		390 (353 - 426)	164.8 (127.9 - 201.6)	1.73 (1.57 - 1.91)	329 (266 - 392)	104.6 (41.4 - 167.8)	1.47 (1.21 - 1.78)	225 (219 - 230)
1991–1994		409 (374 - 443)	234.4 (199.7 - 269.2)	2.34 (2.15 - 2.56)	268 (222 - 313)	93.2 (47.4 - 138.9)	1.53 (1.29 - 1.82)	174 (170 - 179)
1996–1999		348 (323 - 373)	219.8 (194.5 - 245.1)	2.72 (2.52 - 2.94)	262 (226 - 298)	134.0 (97.4 - 170.5)	2.05 (1.78 - 2.36)	128 (124 - 132)
2001–2006		288 (271 - 306)	187.7 (170.2 - 205.3)	2.86 (2.68 - 3.06)	223 (198 - 247)	122.0 (97.4 - 146.6)	2.21 (1.98 - 2.48)	101 (98 - 104)
2006–2011		240 (225 - 254)	168.9 (154.1 - 183.7)	3.38 (3.16 - 3.62)	170 (151 - 190)	99.3 (79.7 - 119.0)	2.40 (2.13 - 2.71)	71 (69 - 73)
Change p.a. (%)		-2.27			-1.75			-4.85
P Trend		<0.01	0.28	<0.01	0.01	0.53	<0.01	<0.01
1981–1984	Lung cancer	88 (69 - 107)	43.4 (24.4 - 62.4)	1.98 (1.58 - 2.47)	24 (6 - 41)	-21.0 (-39.1 - -2.9)	0.53 (0.25 - 1.14)	45 (42 - 47)
1986–1989		86 (69 - 103)	46.6 (29.4 - 63.8)	2.17 (1.77 - 2.67)	45 (21 - 68)	4.8 (-18.7 - 28.2)	1.12 (0.66 - 1.90)	40 (37 - 42)
1991–1994		100 (82 - 117)	65.6 (48.2 - 83.1)	2.94 (2.44 - 3.53)	44 (23 - 64)	9.6 (-11.4 - 30.6)	1.28 (0.79 - 2.08)	34 (32 - 36)
1996–1999		94 (81 - 107)	66.3 (53.2 - 79.4)	3.36 (2.89 - 3.91)	64 (44 - 83)	35.5 (15.9 - 55.1)	2.26 (1.66 - 3.10)	28 (26 - 30)
2001–2006		77 (68 - 86)	49.3 (40.1 - 58.6)	2.79 (2.45 - 3.17)	39 (28 - 50)	11.4 (0.5 - 22.2)	1.41 (1.07 - 1.87)	28 (26 - 29)
2006–2011		71 (63 - 79)	50.7 (42.6 - 58.8)	3.47 (3.06 - 3.94)	50 (39 - 61)	29.4 (17.9 - 40.9)	2.43 (1.92 - 3.08)	21 (19 - 22)
Change p.a. (%)		-0.74			1.64			-2.77
P Trend		0.07	0.87	0.07	0.3	0.07	0.13	<0.01
1981–1984	Non-lung cancer	132 (110 - 154)	29.1 (7.1 - 51.1)	1.28 (1.08 - 1.52)	150 (96 - 204)	47.0 (-7.1 - 101.2)	1.46 (1.01 - 2.09)	103 (99 - 107)
1986–1989		134 (114 - 153)	29.6 (9.7 - 49.6)	1.29 (1.11 - 1.49)	141 (102 - 181)	37.6 (-2.5 - 77.7)	1.36 (1.02 - 1.81)	104 (100 - 108)
1991–1994		149 (129 - 169)	42.4 (22.5 - 62.3)	1.40 (1.22 - 1.60)	110 (80 - 141)	3.7 (-27.3 - 34.8)	1.04 (0.78 - 1.37)	107 (103 - 110)
1996–1999		146 (130 - 162)	45.2 (29.3 - 61.2)	1.45 (1.30 - 1.62)	135 (108 - 161)	33.7 (6.8 - 60.6)	1.33 (1.09 - 1.63)	101 (97 - 104)
2001–2006		164 (151 - 177)	61.4 (48.0 - 74.8)	1.60 (1.47 - 1.74)	125 (106 - 143)	21.8 (3.1 - 40.5)	1.21 (1.04 - 1.41)	103 (100 - 106)
2006–2011		143 (132 - 154)	51.3 (40.0 - 62.7)	1.56 (1.44 - 1.69)	99 (84 - 113)	6.8 (-7.9 - 21.5)	1.07 (0.92 - 1.25)	92 (89 - 94)
Change p.a. (%)		0.57			-1.22			-0.35
P Trend		0.36	0.04	0.01	0.08	0.16	0.12	0.11
1981–1984	Respiratory disease	82 (65 - 99)	47.1 (30.2 - 64.1)	2.36 (1.90 - 2.93)	63 (15 - 112)	28.7 (-19.9 - 77.2)	1.83 (0.85 - 3.95)	35 (32 - 37)
1986–1989		62 (47 - 77)	33.9 (19.1 - 48.8)	2.21 (1.72 - 2.83)	87 (50 - 123)	58.6 (21.9 - 95.3)	3.09 (2.01 - 4.74)	28 (26 - 30)
1991–1994		52 (38 - 66)	31.0 (17.0 - 44.9)	2.45 (1.86 - 3.23)	54 (27 - 82)	33.1 (5.2 - 61.0)	2.55 (1.52 - 4.29)	21 (20 - 23)
1996–1999		50 (40 - 61)	29.6 (19.3 - 40.0)	2.43 (1.96 - 3.02)	38 (23 - 53)	17.1 (1.9 - 32.4)	1.83 (1.22 - 2.75)	21 (19 - 22)
2001–2006		51 (43 - 58)	32.3 (24.3 - 40.4)	2.78 (2.35 - 3.29)	31 (21 - 42)	13.1 (2.7 - 23.4)	1.72 (1.23 - 2.41)	18 (17 - 19)
2006–2011		44 (38 - 51)	31.2 (24.7 - 37.7)	3.38 (2.87 - 3.98)	30 (20 - 40)	17.0 (7.1 - 26.8)	2.29 (1.64 - 3.21)	13 (12 - 14)
Change p.a. (%)		-2.14			-3.83			-3.34
P Trend		0.02	0.21	0.02	0.04	0.18	0.36	<0.01
1981–1984	Diabetes	32 (20 - 44)	24.5 (12.6 - 36.5)	4.49 (2.99 - 6.75)	33 (6 - 60)	25.8 (-1.5 - 53.0)	4.67 (2.01 - 10.86)	7 (6 - 8)
1986–1989		44 (32 - 56)	38.2 (26.5 - 49.8)	7.65 (5.63 - 10.39)	31 (10 - 52)	25.1 (4.3 - 45.9)	5.37 (2.69 - 10.72)	6 (5 - 7)
1991–1994		55 (42 - 69)	50.4 (37.1 - 63.7)	11.34 (8.50 - 15.13)	23 (9 - 36)	17.6 (4.1 - 31.1)	4.61 (2.48 - 8.56)	5 (4 - 6)
1996–1999		62 (52 - 73)	56.1 (45.4 - 66.7)	10.07 (8.10 - 12.52)	39 (25 - 53)	32.5 (18.3 - 46.6)	6.25 (4.23 - 9.23)	6 (5 - 7)
2001–2006		67 (58 - 75)	59.8 (51.3 - 68.3)	9.59 (8.13 - 11.31)	59 (46 - 71)	51.5 (39.2 - 63.9)	8.40 (6.63 - 10.64)	7 (6 - 8)
2006–2011		56 (49 - 62)	49.9 (43.0 - 56.9)	9.93 (8.40 - 11.74)	48 (37 - 59)	42.2 (31.4 - 53.0)	8.55 (6.64 - 10.99)	6 (5 - 6)
Change p.a. (%)		2.06			2.63			-0.16
P Trend		0.11	0.1	0.27	0.08	0.07	0.01	1
1981–1984	Unintentional injury	95 (79 - 110)	46.1 (30.1 - 62.0)	1.95 (1.63 - 2.33)	62 (39 - 86)	14.0 (-9.9 - 37.9)	1.29 (0.88 - 1.89)	48 (45 - 51)
1986–1989		82 (71 - 94)	33.6 (21.8 - 45.4)	1.69 (1.46 - 1.97)	56 (38 - 74)	7.8 (-10.3 - 25.9)	1.16 (0.84 - 1.60)	48 (46 - 51)
1991–1994		78 (66 - 89)	37.9 (26.0 - 49.7)	1.95 (1.65 - 2.29)	36 (23 - 48)	-4.2 (-16.9 - 8.6)	0.90 (0.63 - 1.28)	40 (37 - 43)
1996–1999		72 (62 - 82)	41.7 (31.3 - 52.0)	2.37 (2.02 - 2.79)	39 (28 - 50)	8.8 (-2.4 - 20.1)	1.29 (0.96 - 1.73)	30 (28 - 33)
2001–2006		54 (48 - 60)	26.1 (19.7 - 32.5)	1.94 (1.70 - 2.21)	35 (27 - 43)	7.1 (-0.7 - 15.0)	1.26 (1.00 - 1.58)	28 (26 - 30)
2006–2011		53 (47 - 58)	26.7 (20.5 - 32.9)	2.03 (1.78 - 2.32)	22 (16 - 28)	-3.4 (-9.6 - 2.8)	0.87 (0.66 - 1.14)	26 (24 - 28)
Change p.a. (%)		-2.32			-3.48			-2.83
P Trend		<0.01	0.06	0.49	0.01	0.3	0.53	<0.01
1981–1984	Suicide	14 (8 - 19)	-3.2 (-8.8 - 2.4)	0.81 (0.54 - 1.22)	13 (4 - 23)	-3.6 (-13.3 - 6.1)	0.78 (0.38 - 1.64)	17 (15 - 19)
1986–1989		17 (12 - 23)	-6.0 (-12.0 - 0.0)	0.74 (0.53 - 1.04)	18 (9 - 27)	-5.3 (-14.8 - 4.3)	0.77 (0.46 - 1.30)	23 (21 - 26)
1991–1994		23 (17 - 28)	-2.0 (-8.2 - 4.1)	0.92 (0.70 - 1.20)	18 (9 - 26)	-6.9 (-15.9 - 2.1)	0.72 (0.43 - 1.19)	25 (22 - 27)
1996–1999		40 (32 - 47)	13.5 (5.4 - 21.7)	1.52 (1.23 - 1.89)	20 (12 - 28)	-6.2 (-14.4 - 2.0)	0.76 (0.51 - 1.15)	26 (24 - 28)
2001–2006		28 (24 - 32)	8.2 (3.6 - 12.7)	1.41 (1.19 - 1.68)	19 (14 - 25)	-0.3 (-5.9 - 5.3)	0.99 (0.74 - 1.32)	20 (18 - 21)
2006–2011		27 (23 - 31)	8.3 (3.9 - 12.7)	1.45 (1.22 - 1.73)	17 (12 - 21)	-1.8 (-6.5 - 3.0)	0.90 (0.68 - 1.20)	18 (17 - 20)
Change p.a. (%)		2.52			0.63			-0.07
P Trend		0.1	0.03	0.05	0.66	0.16	0.12	0.8

**Table 2:** 1–74-year-old female all-cause and cause-specific standardized mortality rates (per 100,000), standardized rate differences (per 100,000) and standardized rate ratios

Females		Total Māori			Total Pacific			European/Other
Follow-up period	Mortality	Standardized rate (per 100,000)	Standardized rate difference (per 100,000)	Standardized rate ratio	Standardized rate (per 100,000)	Standardized rate difference (per 100,000)	Standardized rate ratio	Standardized rate (per 100,000)
1981–1984	All-cause	692 (642 - 742)	389.5 (339.0 - 440.0)	2.29 (2.12 - 2.47)	389 (315 - 462)	86.6 (12.7 - 160.4)	1.29 (1.06 - 1.56)	302 (296 - 309)
1986–1989		626 (585 - 668)	347.3 (305.5 - 389.1)	2.25 (2.09 - 2.41)	425 (359 - 491)	146.0 (79.4 - 212.7)	1.52 (1.30 - 1.78)	279 (273 - 285)
1991–1994		612 (575 - 649)	365.4 (328.1 - 402.7)	2.48 (2.33 - 2.65)	386 (336 - 436)	139.7 (89.6 - 189.8)	1.57 (1.37 - 1.79)	246 (241 - 252)
1996–1999		597 (566 - 629)	382.4 (350.8 - 414.0)	2.78 (2.63 - 2.94)	393 (352 - 434)	178.1 (136.9 - 219.3)	1.83 (1.64 - 2.03)	215 (210 - 220)
2001–2006		571 (548 - 593)	363.7 (341.0 - 386.4)	2.76 (2.64 - 2.88)	356 (328 - 383)	148.7 (120.9 - 176.5)	1.72 (1.59 - 1.86)	207 (203 - 211)
2006–2011		475 (457 - 494)	300.7 (282.1 - 319.4)	2.72 (2.61 - 2.84)	348 (324 - 372)	173.0 (148.8 - 197.1)	1.99 (1.85 - 2.14)	175 (171 - 178)
Change p.a. (%)		-1.16			-0.59			-2.04
P Trend		0.01	0.11	0.03	0.02	0.13	0.02	<0.01
1981–1984	CVD	304 (270 - 338)	185.4 (151.2 - 219.7)	2.57 (2.28 - 2.88)	158 (112 - 203)	39.2 (-6.9 - 85.2)	1.33 (0.99 - 1.78)	118 (114 - 122)
1986–1989		258 (231 - 285)	162.3 (134.7 - 190.0)	2.70 (2.41 - 3.02)	167 (121 - 213)	70.9 (24.8 - 117.0)	1.74 (1.32 - 2.30)	96 (92 - 99)
1991–1994		228 (204 - 252)	154.5 (130.5 - 178.6)	3.10 (2.77 - 3.47)	149 (116 - 181)	74.9 (42.3 - 107.5)	2.02 (1.62 - 2.52)	74 (71 - 76)
1996–1999		201 (182 - 219)	148.7 (129.9 - 167.5)	3.87 (3.49 - 4.29)	129 (106 - 152)	77.3 (53.8 - 100.7)	2.49 (2.07 - 3.00)	52 (50 - 54)
2001–2006		169 (157 - 182)	124.7 (111.9 - 137.4)	3.79 (3.49 - 4.13)	109 (93 - 125)	64.1 (48.1 - 80.0)	2.43 (2.09 - 2.83)	45 (43 - 46)
2006–2011		124 (115 - 134)	93.8 (83.9 - 103.8)	4.07 (3.71 - 4.46)	91 (78 - 103)	60.2 (47.7 - 72.8)	2.97 (2.57 - 3.43)	31 (29 - 32)
Change p.a. (%)		-3.06			-2.18			-5.02
P Trend		<0.01	<0.01	<0.01	<0.01	0.64	<0.01	<0.01
1981–1984	Lung cancer	56 (41 - 70)	43.7 (29.0 - 58.3)	4.62 (3.49 - 6.12)				12 (11 - 13)
1986–1989		55 (43 - 67)	40.7 (28.7 - 52.8)	3.93 (3.09 - 4.99)	19 (4 - 34)	5.1 (-9.6 - 19.9)	1.37 (0.63 - 2.97)	14 (13 - 15)
1991–1994		58 (47 - 69)	43.0 (31.9 - 54.0)	3.78 (3.08 - 4.65)	12 (4 - 21)	-3.1 (-11.8 - 5.7)	0.80 (0.40 - 1.62)	15 (14 - 17)
1996–1999		69 (58 - 80)	53.2 (42.1 - 64.2)	4.35 (3.64 - 5.20)	21 (11 - 32)	5.5 (-5.1 - 16.0)	1.34 (0.82 - 2.21)	16 (15 - 17)
2001–2006		74 (66 - 82)	57.0 (48.6 - 65.3)	4.31 (3.80 - 4.90)	26 (18 - 33)	8.6 (0.9 - 16.3)	1.50 (1.11 - 2.03)	17 (16 - 18)
2006–2011		65 (58 - 71)	48.5 (41.8 - 55.2)	4.01 (3.56 - 4.52)	20 (15 - 26)	4.3 (-1.5 - 10.0)	1.26 (0.95 - 1.68)	16 (15 - 17)
Change p.a. (%)		1.02			1.5			1.2
P Trend		0.17	0.24	0.79	0.04	0.06	0.42	0.02
1981–1984	Non-lung cancer	127 (107 - 147)	28.5 (8.6 - 48.5)	1.29 (1.10 - 1.51)	95 (59 - 130)	-3.8 (-39.7 - 32.0)	0.96 (0.66 - 1.40)	99 (95 - 102)
1986–1989		122 (105 - 139)	25.6 (8.3 - 42.9)	1.27 (1.10 - 1.46)	105 (72 - 138)	8.4 (-24.9 - 41.6)	1.09 (0.79 - 1.49)	97 (93 - 100)
1991–1994		123 (107 - 138)	29.1 (13.3 - 44.8)	1.31 (1.15 - 1.49)	90 (67 - 113)	-4.0 (-27.1 - 19.2)	0.96 (0.74 - 1.24)	94 (90 - 97)
1996–1999		139 (125 - 153)	49.8 (35.0 - 64.6)	1.56 (1.40 - 1.74)	115 (92 - 138)	26.2 (3.0 - 49.3)	1.29 (1.06 - 1.58)	89 (86 - 92)
2001–2006		139 (128 - 150)	53.7 (42.8 - 64.6)	1.63 (1.50 - 1.77)	100 (86 - 114)	14.6 (0.2 - 29.1)	1.17 (1.01 - 1.36)	85 (83 - 88)
2006–2011		137 (128 - 147)	60.8 (50.9 - 70.8)	1.80 (1.66 - 1.94)	114 (101 - 128)	37.9 (24.3 - 51.6)	1.50 (1.33 - 1.69)	77 (74 - 79)
Change p.a. (%)		0.49			0.6			-0.9
P Trend		0.06	<0.01	<0.01	0.22	0.05	0.04	<0.01
1981–1984	Respiratory disease	62 (47 - 76)	47.7 (33.3 - 62.1)	4.43 (3.44 - 5.70)	21 (6 - 36)	7.0 (-8.1 - 22.2)	1.50 (0.73 - 3.12)	14 (13 - 15)
1986–1989		67 (53 - 82)	51.6 (37.0 - 66.1)	4.31 (3.41 - 5.45)	34 (18 - 49)	18.0 (2.8 - 33.2)	2.16 (1.36 - 3.42)	16 (14 - 17)
1991–1994		57 (45 - 69)	43.3 (31.2 - 55.4)	4.12 (3.28 - 5.17)	25 (12 - 38)	11.1 (-2.5 - 24.6)	1.80 (1.04 - 3.10)	14 (13 - 15)
1996–1999		52 (43 - 62)	38.7 (29.0 - 48.4)	3.82 (3.12 - 4.68)	15 (8 - 23)	1.5 (-6.3 - 9.4)	1.11 (0.66 - 1.86)	14 (13 - 15)
2001–2006		58 (51 - 66)	43.8 (36.3 - 51.4)	4.01 (3.47 - 4.64)	19 (13 - 25)	4.4 (-1.8 - 10.6)	1.31 (0.94 - 1.81)	15 (14 - 16)
2006–2011		51 (44 - 57)	38.3 (31.9 - 44.8)	4.16 (3.59 - 4.81)	20 (14 - 26)	7.5 (1.6 - 13.5)	1.62 (1.19 - 2.20)	12 (11 - 13)
Change p.a. (%)		-0.81			-1.4			-0.32
P Trend		0.1	0.09	0.37	0.46	0.66	0.4	0.17
1981–1984	Diabetes	34 (21 - 46)	29.2 (16.8 - 41.7)	7.84 (5.17 - 11.89)	33 (7 - 59)	28.8 (3.0 - 54.7)	7.75 (3.47 - 17.32)	4 (4 - 5)
1986–1989		34 (23 - 44)	29.2 (18.8 - 39.6)	7.70 (5.40 - 10.98)	24 (9 - 39)	19.7 (4.4 - 34.9)	5.52 (2.86 - 10.65)	4 (4 - 5)
1991–1994		49 (38 - 60)	45.3 (34.5 - 56.0)	13.43 (10.11 - 17.85)	25 (11 - 38)	20.9 (7.4 - 34.4)	6.74 (3.78 - 12.02)	4 (3 - 4)
1996–1999		47 (38 - 57)	44.1 (34.7 - 53.6)	15.98 (12.15 - 21.01)	45 (31 - 59)	42.3 (28.2 - 56.5)	15.36 (10.68 - 22.10)	3 (2 - 4)
2001–2006		45 (38 - 51)	40.9 (34.4 - 47.3)	12.29 (10.07 - 15.01)	43 (33 - 52)	38.9 (29.3 - 48.4)	11.74 (9.01 - 15.30)	4 (3 - 4)
2006–2011		28 (24 - 33)	25.4 (20.9 - 29.9)	9.61 (7.75 - 11.91)	40 (32 - 49)	37.2 (28.5 - 45.9)	13.62 (10.50 - 17.66)	3 (3 - 3)
Change p.a. (%)		0.06			1.75			-2.52
P Trend		0.41	0.47	0.88	0.11	0.1	0.18	0.07
1981–1984	Unintentional injury	25 (18 - 31)	8.1 (1.7 - 14.5)	1.49 (1.14 - 1.96)	15 (2 - 27)	-1.8 (-14.7 - 11.1)	0.89 (0.37 - 2.16)	16 (15 - 18)
1986–1989		27 (20 - 33)	11.1 (4.4 - 17.7)	1.71 (1.31 - 2.22)	16 (9 - 24)	0.6 (-7.2 - 8.3)	1.04 (0.64 - 1.67)	16 (14 - 17)
1991–1994		28 (21 - 34)	14.8 (8.2 - 21.3)	2.14 (1.66 - 2.77)	14 (7 - 21)	1.0 (-6.0 - 8.0)	1.08 (0.65 - 1.78)	13 (12 - 14)
1996–1999		21 (17 - 26)	11.1 (6.3 - 15.9)	2.11 (1.64 - 2.72)	10 (5 - 16)	0.2 (-5.3 - 5.7)	1.02 (0.59 - 1.75)	10 (9 - 11)
2001–2006		22 (18 - 26)	12.9 (9.1 - 16.8)	2.44 (2.00 - 2.98)	7 (4 - 10)	-2.3 (-5.6 - 1.1)	0.75 (0.46 - 1.22)	9 (8 - 10)
2006–2011		18 (15 - 21)	8.6 (5.6 - 11.6)	1.94 (1.60 - 2.34)	9 (6 - 13)	0.1 (-3.6 - 3.7)	1.01 (0.68 - 1.50)	9 (8 - 10)
Change p.a. (%)		-1.36			-2.83			-2.54
P Trend		0.03	0.67	0.21	0.09	0.68	0.59	0.01
1981–1984	Suicide	3 (1 - 5)	-3.5 (-6.0 - -1.0)	0.45 (0.20 - 1.01)				6 (5 - 8)
1986–1989		4 (2 - 6)	-2.5 (-4.8 - -0.1)	0.63 (0.38 - 1.06)				7 (6 - 8)
1991–1994		4 (2 - 7)	-2.2 (-4.7 - 0.3)	0.66 (0.38 - 1.16)	3 (0 - 6)	-3.2 (-6.3 - -0.2)	0.50 (0.20 - 1.24)	6 (5 - 8)
1996–1999		10 (7 - 14)	3.9 (0.6 - 7.1)	1.59 (1.13 - 2.23)	6 (2 - 10)	-0.6 (-4.3 - 3.2)	0.91 (0.49 - 1.70)	7 (6 - 8)
2001–2006		8 (6 - 10)	1.7 (-0.4 - 3.9)	1.26 (0.97 - 1.65)	4 (2 - 6)	-3.0 (-5.2 - -0.7)	0.55 (0.31 - 0.99)	7 (6 - 7)
2006–2011		9 (7 - 11)	3.8 (1.7 - 5.9)	1.71 (1.32 - 2.20)	4 (2 - 6)	-1.1 (-3.1 - 0.8)	0.79 (0.50 - 1.24)	5 (5 - 6)
Change p.a. (%)		4.89			-0.17			-0.47
P Trend		0.02	0.01	0.04	0.2	0.09	0.38	0.16

To calculate the contribution of a given cause of mortality to absolute inequalities in overall mortality, we divided each mortality specific SRD by the all-cause mortality SRD.

### Inequality classification and typology graphs

When assessing changing inequalities in mortality between groups in society, it is desirable to report on both the relative and absolute scales simultaneously, whilst assessing the underlying mortality rates for each respective group. The rate of the ethnic group of interest, SRR and SRD are mathematically interrelated – if one knows the Māori rate and rate ratio, for example, one can calculate the rate difference through simple formula rearrangement. This means that all of the standardized rates, SRR, and SRD for a given mortality can be displayed on the same plot, with the trends over time of the three respective measures summarized by one line.

As such, we applied a visualization technique similar to Blakely et al.’s [21] socioeconomic plots (see Figs. 3 and 4). The log of the rate for either Māori or Pacific peoples are plotted on the x-axis, the log of the SRR (European/Other as reference) on the y-axis, and contour plots for SRDs (again with European/Other as reference). This allows simultaneous assessment of the trends in all of relative and absolute inequality and the ethnic-specific mortality rate for either Māori or Pacific peoples.

All six cohorts were plotted for all-cause mortality and CVD respectively. For the remaining six causes of mortality (non-lung cancer, lung cancer, respiratory, diabetes, suicide, and unintentional injury), the rates in the 1980s, 1990s, and 2000s are averaged to gain statistical stability.

To further aid classification of trends in both mortality rates, SRDs, and SRRs, for each given mortality, the Blakely et al. typology is used to compare Māori and Pacific to European/Other. Each mortality is assigned a label (see Table 3) to denote whether Māori/Pacific mortality rates (denoted by ‘*m’*), absolute inequalities (denoted by ‘*a’*) and relative inequalities (denoted by *‘r’*) are declining (*↓*), stable (-), or increasing (*↑*). As trends change over time and statistical power varies, where there is a significant p-value on the test for linear trend the appropriate component (*‘m,’ ‘a,’* or *‘r’*) of the inequality typology is underlined.

**Table 3:** Inequality classification for each disease-specific standardized mortality rate (per 100,000), standardized rate difference (per 100,000) and standardized rate ratio for Māori and Pacific peoples, 1-74-year-olds, males and females

	Māori				Pacific	
Disease group	Male		Female		Male	Female
All-cause	***m↓*** *a↓* ***r↑***		***m↓*** *a↓* ***r↓***	***m↓*** *a↓* ***r-***	***m↓*** *a↓* ***r↑***	***m↓*** *a↑* ***r↑***
CVD	***m↓*** *a↓* ***r↑***		***m↓ a↓ r↑***	***m↓ a↓ r-***	***m↓*** *a↓* ***r↑***	***m↓*** *a↑* ***r↑***
Lung cancer	*m↑ a↑ r↑*	*m↓ a↓ r-*	*m↑ a↑ r-*		*m↑ a↑ r↑*	*m- a- r-*
Non-lung cancers	*m↑* ***a↑ r↑***		*m↑* ***a↑ r↑***		*m↓ a↓ r↓*	*m↑ a↑ r↑*
Respiratory disease	***m↓*** *a↓* ***r↑***		*m↓ a↓ r-*		***m↓*** *a↓ r↓*	***m↓*** *a↓ r-*
Diabetes	*m↑ a↑ r↑*		*m- a- r↑*		*m↑ a↑* ***r↑***	*m↑ a↑* ***r↑***
Unintentional injury	***m↓*** *a↓ r-*		***m↓*** *a- r↑*		***m↓*** *a↓ r-*	***m↓*** *a- r-*
Suicide	*m↑ a↑ r↑*	*m↓ a↓ r-*	***m↑ a↑ r↑***		*m- a- r-*	*m- a- r-*

Typologies are assigned according to underlying trends in mortality rates, taking the study period as a whole. Where the trend on the typology plot clearly changes over time, two typologies are assigned to a given mortality. A bold underlined component of the typology indicates that particular trend was found to be significant at the 95% level (*p* < 0.05)

The least desirable pattern, from a health perspective, is that of increasing mortality rates, increasing absolute (SRD) and relative inequalities (SRR), which would be labeled as ‘*m↑ a↑ r↑’.* In terms of a trend line on the typology plots (Figs. 3 and 4), a compass analogy is used to allow interpretation across an array of trend line directions. An increasing mortality rate would appear as a shift to the east (right) on the horizontal (x-axis). Where relative inequalities have increased, there is a shift north (upwards) with respect to the vertical axis (y-axis), and an increase in absolute inequalities results in a shift across the SRD contour lines in mainly northeast (right and up) direction.

## Results

### All-cause mortality

For each ethnic group there was a fall in all-cause mortality rates over the study period. Before analyzing any changes in inequality, we first considered the annual percentage change in all-cause mortality over the study period, for each ethnic group. For males, the annual percentage change for European/Other was -2.65%; whereas, for Māori it was -1.21% and for Pacific peoples it was -1.19%, with all three test for linear trends being statistically significant (Table 1). For females there is a similar ranking – European/Other falling most quickly (-2.04%) followed by Māori (-1.16%), with the Pacific female rate only falling by -0.59%.

**Fig. 1:**
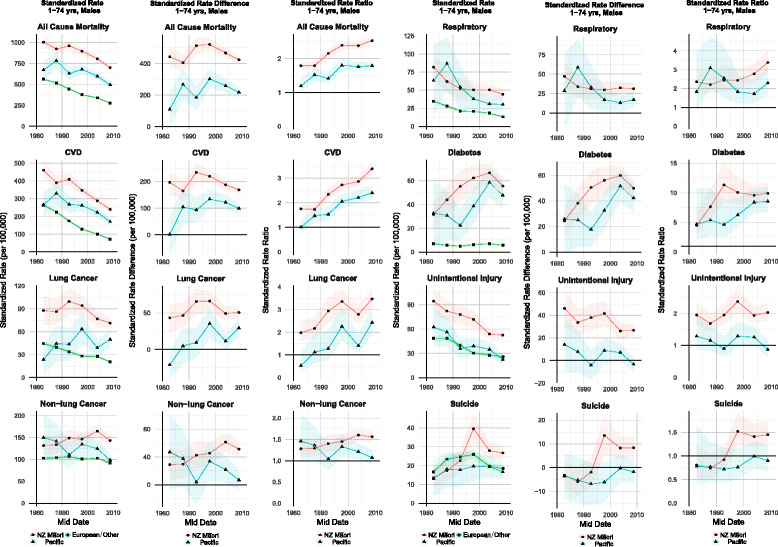
Trends in *male* 1–74 year old all-cause and cause-specific standardized mortality rates (per 100,000), absolute standardized rate differences (per 100,000) and rate ratios for Māori and Pacific compared to European/Other. Shaded area around trends line denotes 95% confidence interval. Data available at: https://nzcms-ct-data-explorer.shinyapps.io/version8/

For Māori males, there was no statistically significant change in rates over the first three follow-up periods (1981–1984 to 1991–1994); whereas, in the final four follow-up periods (1991–1994 to 2006–2011) there was a significant downward trend in mortality from 960 (per 100,000) to 701 (per 100,000). Pacific males showed an overall similar pattern to Māori in all-cause rates – a period of no significant reduction in mortality followed by a downward trend, in this case the final three follow-up periods (Fig. 1).

Absolute inequalities in all-cause mortality for Māori males (European/Other) showed a statistically significant reduction in SRDs in the three most recent cohorts, from 521 (per 100,000) in 1996–1999 to 424 (per 100,000) in 2006–2011 (Table 1 and Fig. 1). In contrast, relative inequalities in all-cause mortality for Māori compared to European/Other increased from 1.79 in 1981–1984 to 2.53 in 2006–2011.

Figure 3 shows the holistic inequality plot for Māori and Pacific males, providing a summary of the trends in SMRs, SRDs, and SRRs simultaneously, with the arrowhead denoting the most recent cohort of data. For Māori males, rates in all-cause mortality (red line) have decreased shifting to the west (left) on the x-axis; relative inequalities have increased (a shift northwards with respect to the y-axis); and there has been little change to absolute inequalities (with little/no movement across the dotted contour lines). The trend was similar for Pacific males and the pattern for both ethnic groups was a ‘*m↓ a↓ r↑’* typology.

**Fig. 2:**
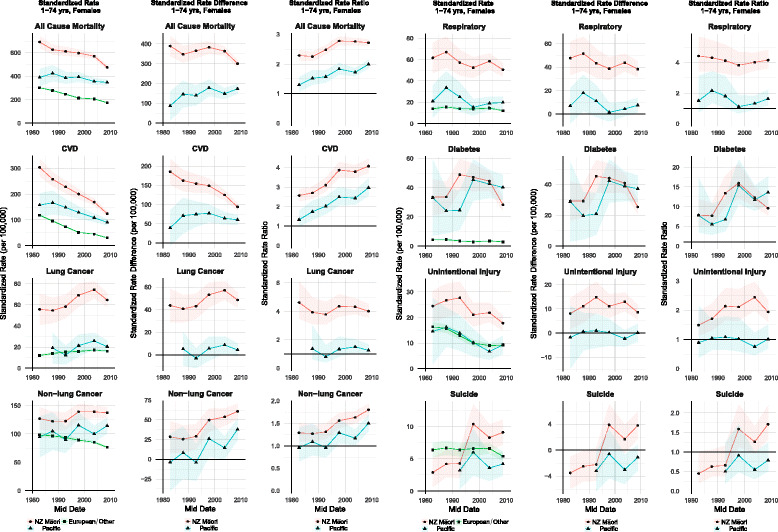
Trends in *female* 1–74 year old all-cause and cause-specific standardized mortality rates (per 100,000), absolute standardized rate differences (per 100,000) and rate ratios for Māori, Pacific and European/Other ethnic groups. Shaded area around trends line denotes 95% confidence interval. Data available at: https://nzcms-ct-data-explorer.shinyapps.io/version8/

Figure 4 shows the inequality typology plot for females. With a consistent decline in the female European/Other mortality rate (Fig. 2), the slower decline evident in the middle cohorts for Māori (flatter trend line in Fig. 2 and less of a westerly movement on Fig. 4) resulted in a slight increase in absolute inequality between 1986–1989 (347 per 100,000) and 1996–1999 (382 per 100,000). There was then a fall to 301 (per 100,000) in 2006–2011 represented in a movement towards the 320 SRD contour on Fig. 4.

The SRR for Māori females is higher at the end of the study period (2.72) than at the beginning (2.29), with no significant change in the three most recent cohorts. In recent cohorts, the trends described above have resulted in the typology line bending round in a westerly direction crossing the 320 SRD contour line (reducing Māori mortality rate, falling absolute inequality, and a slight fall in relative inequalities) – a marginal ‘*m↓ a↓ r↓’* or ‘*m↓ a↓ r-‘*typology.

For Pacific females, the lower annual percentage change reduction in all-cause mortality than European/Other and Māori is reflected in an increase in the SRDs over the study period, from 87 to 173 (per 100,000). Relative inequalities also increase from 1.29 (1981–1984) to 1.99 (2006–2011). This is reflected in a general northerly movement on the inequality typology plot (Fig. 4). The line, having moved completely across the gap between the 80 SRD and 160 SRD contours (an increase in absolute inequality), only moves slightly westwards (a slow fall in the Pacific all-cause rate) and shifts north (indicating increasing relative inequalities). There is some noise evident in the trend, due to smaller numbers; however, the more general trend across cohorts suggests a ‘*m↓ a↑ r↑’* typology.

### Major diseases contributing to the trends in inequalities

#### General transition

CVD still remains the largest specific disease contributor to absolute inequalities in all-cause mortality by ethnicity (highest SRDs are for CVD; arrow head for most recent cohort on holistic inequality plots for CVD positioned more north(east) than other mortalities of interest). However, there is a transition evident: away from CVD and towards cancer as the emerging important contributor to absolute inequalities.

#### Contribution of CVD

Overall, there was a decline in CVD rates for both males and females (Tables 1 and 2). For males, the annual percentage change over the entire 30 years for European/Other (-4.81%) exhibits a faster rate of decline than both Māori (-2.23%) and Pacific (-1.71%). This ethnic-specific pattern is also evident for females: European/Other -4.98%; Māori -3.05%; Pacific -2.15%.

In terms of the disease-specific contribution to all-cause absolute inequalities, CVD has remained the main contributor. For Māori males, the SRD accounts for 39% of the all-cause absolute inequality. Its contribution was higher in earlier cohorts, for example 44% in 1991–1994 and 43% in 1981–1984. However, absolute inequality is falling in the three most recent cohorts. Considering relative inequality, absolute inequality, and the male Māori mortality rate concurrently, there is a bend towards a north-west direction (mortality rate falling, SRD beginning to fall, and SRR consistently increasing) – a ‘*m↓ a↓ r↑’* classification.

**Fig. 3:**
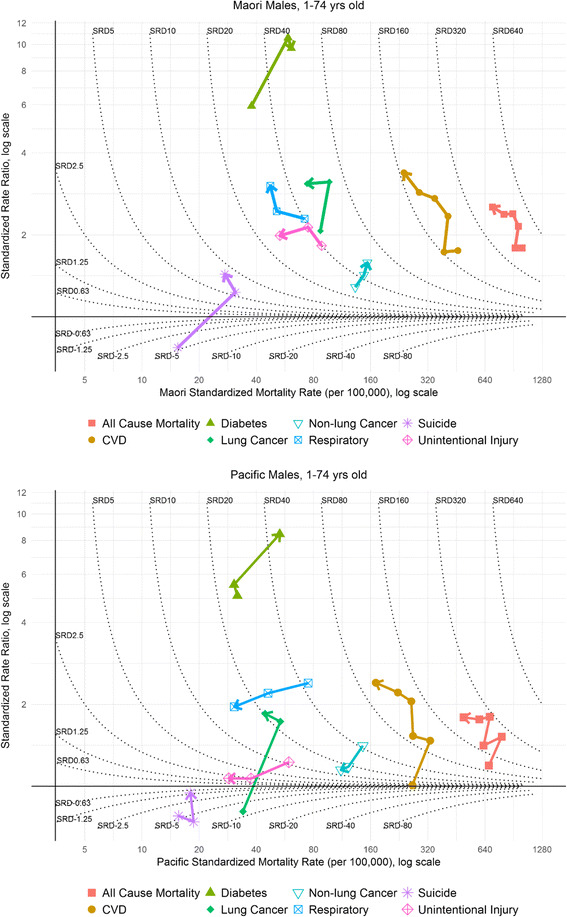
Graphical presentation of Māori and Pacific inequalities and mortality rates for males, 1–74-year-olds 1981–2011 (with SRDs (contour lines) and SRRs (log scale y-axis) compared to European/Other)

For male Pacific peoples, the CVD SRD contributed 45% of the all-cause absolute inequality. Over the first four cohorts, there was a northerly trend on the inequality typology (Fig. 3) – increases in absolute and relative inequalities and little to no change in the mortality rate. In the two most recent cohorts, though, this trend has bent round to a north-westerly direction as a result of a falling mortality rate, falling absolute inequalities and slower increases in relative inequality (classified as ‘*m↓ a↓ r↑’).*

For female Māori, there have been a statistically significant fall in absolute inequalities for CVD. With this reduction in absolute inequalities, the importance of CVD, in terms of its contribution to all-cause absolute inequalities, has declined. In 2006–2011 it contributed 31% of the absolute gap in all-cause mortality between Māori and European/Other; down from 47% in 1981–1984. In the three most recent cohorts there has been a marked reduction in the SRD and a plateauing of the SRR – the line on the inequality typology plot has consequently turned westward, – on the borderline between a ‘*m↓ a↓ r↑’* and ‘*m↓ a↓ r-‘*classification.

Even though the Pacific SRD for CVD was consistently lower than Māori, absolute inequalities have increased. The SRDs in more recent cohorts suggest that absolute inequalities have plateaued, however. The contribution of CVD, to absolute inequalities, has recently declined – peaking at 53% in 1991–1994 and falling to 35% in 2006–2011.

#### Cancer patterns – particularly for females

Cancer has become a more important contributor to absolute inequalities for Māori, especially for females. For example, the mortality rate for Māori females has a much later peak, during 2001–2006, than for Māori males, during 1991–1994 (Figs. 1 and 2).

In particular, absolute and relative inequalities for lung cancer in Māori females are large and greater than for Pacific. The contribution of lung cancer to all-cause absolute inequality for Māori females is 16% and less than 1% for Pacific. There is a similar pattern for males.

**Fig. 4:**
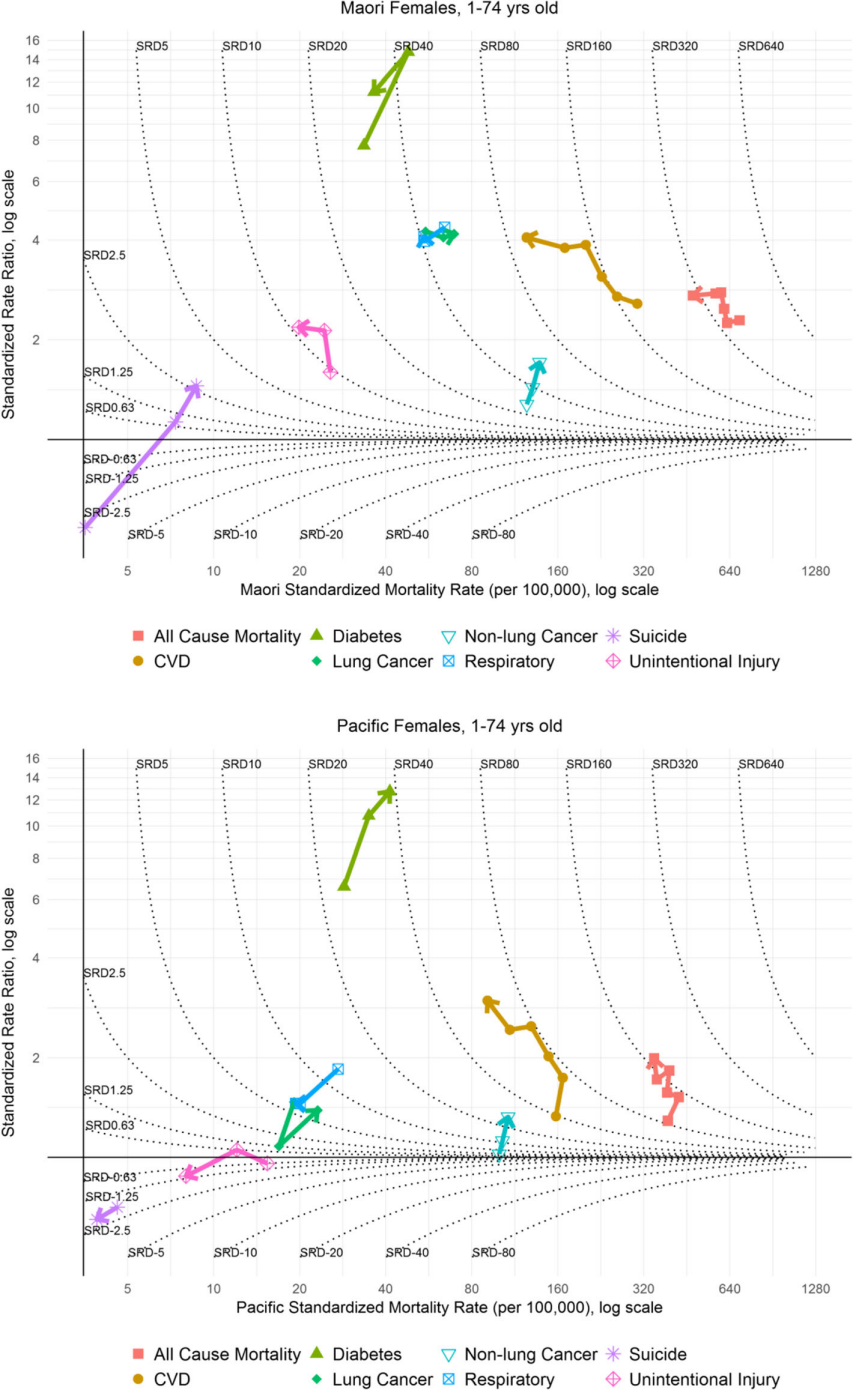
Graphical presentation of Māori and Pacific inequalities and mortality rates for females, 1–74 years olds 1981–2011 (with SRDs (contour lines) and SRRs (log scale y-axis) compared to European/Other)

Considering both inequality measures and the standardized mortality rate concurrently, there is an eastward movement for the lung cancer typology line, from the 1980s to 2000s, for Māori females (Fig. 4), resulting in a summary ‘*m↑ a↑ r-’* typology across the whole study period.

For both Māori and Pacific females, non-lung cancer rates have been increasing. The trends in inequality are similar to lung cancer (Fig. 4) – a general north-east direction and ‘*m↑ a↑ r↑’* classification. In recent cohorts, females of both ethnic groups have a higher mortality rate for non-lung cancer than CVD, but with lower relative and absolute inequalities.

#### Other selected drivers of inequalities

There has been a fall in absolute inequalities in unintentional injury for Māori males, from 45 (per 100,000) to 26 (per 100,000), alongside a falling mortality rate (annual percentage change of -2.28%). The inequality typology plots for unintentional injury exhibit a general westward movement and typology classification of ‘*m↓ a↓ r-’.* But for suicide in Māori, there have been large increasing absolute and relative inequalities, reflected in a general northern movement in the typology plot (Fig. 3).

Diabetes clearly has the highest level of relative inequalities for both Māori and Pacific (Figs. 3 and 4). There was a significant increase in relative inequalities for each ethnic group by sex combination, and there has been a worsening of both relative and absolute inequalities since the beginning of the study period. Pacific peoples’ inequalities from diabetes have been marginally worse than for Māori (moving northeast on Figs. 3 and 4).

## Discussion

### Main findings

The decline in all-cause mortality rates for all ethnic groups in New Zealand is a favourable finding from a public health perspective. Even so, for males during the 1980s and 1990s there was actually a slight increase and stagnation in this pattern for Māori and Pacific. This development, largely driven by CVD, contributed to an increase in absolute inequalities, which are now falling in the three most recent cohorts.

Another main finding is the continuation of substantial relative and absolute inequalities in mortality for both Māori and Pacific in relation to European/Other. Pacific all-cause mortality has been falling at a slower rate than both Māori and European/Other across the whole study period, and particularly for females. This has resulted in increasing absolute inequalities for Pacific females while the opposite has been the case for Māori females across the whole study period.

All-cause relative inequalities are still high and increasing for Māori males and Pacific females. However, for Pacific males and Māori females, there are signs that relative inequality, whilst still high, has not been getting worse.

In terms of the main contributors to inequalities, there appears to be a transition, particularly amongst females, away from CVD and towards cancer, especially non-lung cancer. The steady decline in female European/Other non-lung cancer mortality rates compared to a steady rise in both Māori and Pacific has resulted in rises of both absolute and relative inequality. Furthermore, whilst there are examples of falling absolute inequalities (mainly respiratory disease and unintentional injury) relative inequalities are, on the whole, large (especially diabetes) and increasing.

The human cost associated with these inequalities is striking. Fortunately, many of the diseases driving this inequality can be addressed – as we detail further below.

### Strengths and limitations of this study

The linking of individual census data to mortality records enabled us to analyze 30 years’ worth of mortality trends for the population of an entire country. Having six follow-up periods has now allowed us to have a greater detailed understanding of how trends in both the rate and inequalities are changing over time.

Furthermore, common deficiencies in ethnicity data have been circumvented by using the self-defined ethnicity variable from the Census, and numerator-denominator bias is avoided. The study size is also very large – with 68.9 million years of person time follow-up. However, there are still specific diseases (e.g., for female Pacific peoples for lung cancer and suicide for 1981–1984) where small numbers limited statistical power. Small numbers also limited the extent to which we could consider mortality-specific trends disaggregated by ethnicity and age. Where numbers permitted (for example, all-cause mortality), we found that trends in ethnic-specific standardized mortality rates are similar by age. Furthermore, there were people of a non-European ethnic background included in the European/Other group (i.e., people who do not self-identify as Māori, Pacific, Asian or European). However, these numbers are likely to be small and not have a significant effect on our findings. Future research could assess the sensitivity of our findings to this and build on previous work [12] to identify whether there is heterogeneity in mortality trends within ethnic groups.

### Potential implications for reducing ethnic inequalities

The ethnic inequalities in mortality identified in this paper are so striking that, we argue, a consideration of how they can be reduced has to start with a critical assessment of how society is structured. Macroeconomic and social policy are one such structuring factor. Previous research for New Zealand has indicated that the widening in ethnic inequalities in 1980s and 1990s was contributed to by structural economic changes to liberalize the market economy [9].

Indeed, further risks for widening inequalities may persist if automation and other globalization forces result in increased unemployment and insecure employment among lower-skilled workers [22]. Nevertheless, there can be progress, as seen with more rapid increases in the percentage of Māori and Pacific school leavers with school qualifications [23]. However, faster progress is required if there is to be a significant erosion of the entrenched inequalities in health we identified. Measures such as additional education support, improvements in social housing provision, tax reform (e.g., re-introducing estate (inheritance) taxes, and reducing income taxes for low-income families) and a living wage should all be seriously considered.

Another structural driver of inequalities in mortality is the obesogenic environment [24]. In New Zealand, as with many other high-income countries, a high body mass index is now the largest single cause of health loss [25]. This is also likely to be a driver for inequalities from CVD, diabetes, and some types of cancer for both Māori and Pacific peoples (given much higher burdens of overweight and obesity [26]).

Detailed analysis of both cancer incidence and mortality by Teng et al. [27] has found that ethnic inequalities have actually widened most in the incidence of obesity-related cancers in New Zealand (breast and endometrial cancer in particular). While the New Zealand government has recently adopted an obesity control strategy, there is still scope for more substantive interventions to address obesogenic environments, e.g., Mexican-style taxes on sugary drinks [28], banning junk food marketing to children, and mandatory front of package health rating labeling of food. If such measures were well-designed, and appropriately implemented, they should contribute to reductions in inequalities (e.g., there is some evidence for greater response by lower socioeconomic groups in Mexico to reduced consumption of sugary drink tax after the new tax [28]).

The second most important cause of health loss in New Zealand is from smoking [25]. Furthermore, our results for ethnic inequalities in lung cancer (and to some extent for CVD, and other cancers) are likely to reflect the marked ethnic gradient in tobacco smoking in New Zealand; both currently and as projected into the future [29]. This highlights the potential for further advances in tobacco control; and, indeed the New Zealand government has a smoke-free nation goal for the year 2025.

Recent interventions include annual tobacco tax increases in the years 2010 to 2016, banning point-of-sales displays of tobacco, and a plan to finalize a law requiring the plain packaging for tobacco products. But interventions that may disproportionately benefit Māori may include tax increases (as per New Zealand modeling work [30]) and mass media campaigns designed for a Māori audience [31, 32]. More profound interventions to accelerate progress to the tobacco endgame have also been discussed for New Zealand and elsewhere: a sinking lid on sales [33], tobacco retail outlet reduction [34], and a smoke-free generation (i.e., no one born after the year 2000 is allowed to purchase tobacco [35]). The New Zealand public appear to be broadly supportive of such endgame strategies [36] and these are consistent with international calls to reduce the overall burden and inequalities from the global tobacco epidemic [37].

A further domain for inequality reduction interventions is in accelerating the ongoing declines in CVD risk. Modeling work for New Zealand has suggested that multiple population-wide dietary sodium reduction measures are likely to provide greater per capita health gain for Māori relative to non-Māori [38]. Health services also matter and there remain some gaps in terms of the provision of CVD risk screening in primary care for Māori vs. non-Māori (at 86% vs. 92% coverage respectively) [39]. However the target age-range for such screening for Māori starts at a young age (to assist with equity goals) and there have been successful campaigns to increase preventive CVD pharmacotherapy among Māori (e.g., for statin use [40]).

Finally, another area for health service improvements are addressing the inequalities, or inequities, in receipt of some cancer treatments between ethnic groups in New Zealand [41] and ethnic/racial groups internationally [42]. Some of these inequalities are challenging to address: e.g., higher comorbidities among ethnic groups, which may be perceived as limiting treatment options. But gains are likely to be possible, and will contribute somewhat to reducing (or at least slowing) ethnic inequalities in mortality. Improvements in diabetes care may also be a likely fertile area for policies and actions to reduce ethnic inequalities in mortality.

## Conclusions

In conclusion, for this case study (New Zealand), we found a favorable decline in all-cause mortality rates over time by ethnic group. Large ethnic mortality inequalities are generally stable (or even falling) in absolute terms, but have increased on a relative scale. The cause of death drivers of inequalities are changing over time, away from CVD to cancer and diabetes. To further decrease (absolute, in particular) inequalities, there is still more to gain from CVD mortality reductions, but increasingly acting on the causes and treatments of cancer and diabetes will be required. Cancer and obesity-related illnesses are likely to remain major contributors to health loss and drivers of New Zealand’s stark ethnic inequalities in mortality. To address these inequalities, policymakers need to enhance prevention activities and improve health care delivery with a clear equity focus, and also support wider improvements in educational achievement and socioeconomic position for Māori and Pacific peoples.

## Abbreviations

CVD: Cardiovascular disease
ICD: International classification of disease
NZCMS: New Zealand Census Mortality Study
SRD: Standardized rate difference
SRR: Standardized rate ratio

## References

[1] Moore SP, Antoni S, Colquhoun A, Healy B, Ellison-Loschmann L, Potter JD (2015). Cancer incidence in indigenous people in Australia, New Zealand, Canada, and the USA: a comparative population-based study. Lancet Oncol.

[2] Bartley M (2004). Health inequality: an introduction to theories, concepts and methods.

[3] Axelsson P, Kukutai T and Kippen R. The field of Indigenous health and the role of colonisation and history. J Popul Res. 2016; 33:1-7.

[4] Anderson I, Robson B, Connolly M, Al-Yaman F, Bjertness E, King A, et al. Indigenous and tribal peoples’ health (The Lancet - Lowitja Institute Global Collaboration): a population study. Lancet. 2016; 338(10040):131-157. 10.1016/S0140-6736(16)00345-7 27108232

[5] Sarfati D, Robson B (2015). Equitable cancer control: better data needed for indigenous people. Lancet Oncol.

[6] Tobias M, Blakely T, Matheson D, Rasanathan K, Atkinson J (2009). Changing trends in indigenous inequalities in mortality: lessons from New Zealand. Int. J. Epidemiol..

[7] Harper S, King NB, Meersman SC, Reichman ME, Breen N, Lynch J (2010). Implicit value judgments in the measurement of health inequalities. Milbank Q.

[8] Woodward A, Blakely T (2014). The Healthy Country? A History of Life and Death in New Zealand.

[9] Blakely T, Tobias M, Atkinson J (2008). Inequalities in mortality during and after restructuring of the New Zealand economy: repeated cohort studies. BMJ.

[10] Statistics New Zealand. New Zealand Period Life Tables: 2010-12 2013 [Available from: http://www.stats.govt.nz/browse_for_stats/health/life_expectancy/NZLifeTables_HOTP10-12.aspx]. (Last accessed: 12.1.17)

[11] Paradies Y. Colonisation, racism and indigenous health. J. Popul. Res. 2016; 33:83-96.

[12] Blakely T, Richardson K, Young J, Callister P, Didham R (2009). Does mortality vary between Pacific groups In New Zealand? Estimating Samoan, Cook Island Maori, Tongan and Niuean mortality rates using hierarchical Bayesian modelling. NZ Med J.

[13] Braveman P (2006). Health disparities and health equity: concepts and measurement. Annu Rev Public Health.

[14] Nazroo JY (2003). The structuring of ethnic inequalities in health: economic position, racial discrimination, and racism. Am J Public Health.

[15] Mir G, Salway S, Kai J, Karlsen S, Bhopal R, Ellison GT (2013). Principles for research on ethnicity and health: the Leeds Consensus Statement. Eur. J. Public Health.

[16] Cormack D and McLeod M. Improving and maintaining quality in ethnicity data collections in the health and disability sector: Te Rōpū Rangahau Hauora a Eru Pōmare, Wellington School of Medicine and Health Sciences, University of Otago (Wellington); 2010.

[17] Boyd M, Atkinson J, Blakely T (2016). Ethnic counts on mortality, NZ Cancer Registry and census data: 2006-2011. NZ Med J.

[18] Blakely T, Salmond C (2002). Probabilistic record linkage and a method to calculate the positive predictive value. Int J Epidemiol.

[19] Disney G, Teng A, Atkinson J and Blakely T. New Zealand Census Mortality and CancerTrends Study Data Explorer. 2016. Available from: https://nzcms-ct-data-explorer.shinyapps.io/version8/. (last accessed 13.1.17).

[20] Statistics New Zealand. Understanding and working with ethnicity data. [Available from: http://www.stats.govt.nz/browse_for_stats/population/census_counts/review-measurement-of-ethnicity/papers.aspx] 2005. (last accessed 13.1.17)

[21] Blakely T, Disney G, Atkinson J, Teng A, Mackenbach J. A typology for charting socio-economic mortality gradients: “go south-west”. Epidemiology. 2017. http://journals.lww.com/epidem/Abstract/publishahead/A_typology_for_charting_socioeconomic_mortality.98856.aspx. 10.1097/EDE.0000000000000671 28394874

[22] Standing G (2011). The Precariat: The New Dangerous Class.

[23] Ministry of Education. More young people with NCEA Level 2. [Available from: http://www.education.govt.nz/ministry-of-education/government-education-initiatives/better-public-services/more-young-people-with-ncea-level-2/]. 2014. (last accessed 13.1.17).

[24] Lake A, Townshend T (2006). Obesogenic environments: exploring the built and food environments. J R Soc Promot Heal.

[25] Forouzanfar M, Alexander L, Anderson H, Bachman V, Biryukov S, GBD 2013 Risk Factors Collaborators (2015). Global, regional, and national comparative risk assessment of 79 behavioural, environmental and occupational, and metabolic risks or clusters of risks in 188 countries, 1990-2013: a systematic analysis for the Global Burden of Disease Study 2013. Lancet.

[26] Ministry of Health. Annual Update of Key Results 2014/15: New Zealand Health Survey. Wellington: Ministry of Health. [Available from: http://www.health.govt.nz/publication/annual-update-key-results-2014-15-new-zealand-health-survey]. 2015. (last accessed 13.1.17)

[27] Teng A, Atkinson J, Disney G, Wilson N, Sarfati D, McLeod M (2016). Ethnic inequalities in cancer incidence and mortality: census-linked cohort studies with 87 million years of person-time follow-up. BMC Cancer.

[28] Colchero MA, Popkin BM, Rivera JA, Ng SW (2016). Beverage purchases from stores in Mexico under the excise tax on sugar sweetened beverages: observational study. BMJ.

[29] van der Deen FS, Ikeda T, Cobiac L, Wilson N, Blakely T (2014). Projecting future smoking prevalence to 2025 and beyond in New Zealand using smoking prevalence data from the 2013 Census. N. Z. Med. J..

[30] Blakely T, Cobiac LJ, Cleghorn CL, Pearson AL, van der Deen FS, Kvizhinadze G (2015). Health, health inequality, and cost impacts of annual increases in tobacco tax: Multistate life table modeling in New Zealand. PLoS Med.

[31] Wilson N, Grigg M, Graham L, Cameron G (2005). The effectiveness of television advertising campaigns on generating calls to a national Quitline by Maori. Tob Control.

[32] Grigg M, Waa A, Bradbrook SK (2008). Response to an indigenous smoking cessation media campaign - it’s about whanau. Aust N Z J Public Health.

[33] Wilson N, Thomson GW, Edwards R, Blakely T (2013). Potential advantages and disadvantages of an endgame strategy: a ‘sinking lid’ on tobacco supply. Tob Control.

[34] Pearson AL, van der Deen FS, Wilson N, Cobiac L, Blakely T (2014). Theoretical impacts of a range of major tobacco retail outlet reduction interventions: modelling results in a country with a smoke-free nation goal. Tob Control.

[35] Berrick AJ (2013). The tobacco-free generation proposal. Tob Control.

[36] Edwards R, Wilson N, Peace J, Weerasekera D, Thomson GW and Gifford H. Support for a tobacco endgame and increased regulation of the tobacco industry among New Zealand smokers: results from a National Survey. Tobacco Control. 2012;[E-publication 27 April]. 10.1136/tobaccocontrol-2011-050324 22535362

[37] Beaglehole R, Bonita R, Yach D, Mackay J, Reddy KS (2015). A tobacco-free world: a call to action to phase out the sale of tobacco products by 2040. Lancet.

[38] Nghiem N, Blakely T, Cobiac LJ, Pearson AL, Wilson N (2015). Health and economic impacts of eight different dietary salt reduction interventions. PLoS One.

[39] Ministry of Health. How is my DHB performing? 2015/16 (Quarter 2 October–December). 2016. [Available from: http://www.health.govt.nz/new-zealand-health-system/health-targets/how-my-dhb-performing/]. (last accessed 13.1.17)

[40] Norris P, Horsburgh S, Becket G, Keown S, Arroll B, Lovelock K (2014). Equity in statin use in New Zealand. J. Prim. Health Care.

[41] Hill S, Sarfati D, Robson B, Blakely T (2013). Indigenous inequalities in cancer: what role for health care?. Aust. N. Z. J. Surg..

[42] Smedley B, Stith A, Nelson A (2002). Unequal Treatment: Confronting Racial and Ethnic Disparities in Health Care.

